# Pathogenic *Escherichia coli* in Dogs Reveals the Predominance of ST372 and the Human-Associated ST73 Extra-Intestinal Lineages

**DOI:** 10.3389/fmicb.2020.00580

**Published:** 2020-04-21

**Authors:** Charlotte Valat, Antoine Drapeau, Stéphanie Beurlet, Véronique Bachy, Henri-Jean Boulouis, Raphaëlle Pin, Géraldine Cazeau, Jean-Yves Madec, Marisa Haenni

**Affiliations:** ^1^Unité Antibiorésistance et Virulence Bactériennes, ANSES Laboratoire de Lyon – Université de Lyon, Lyon, France; ^2^Vebio, Arcueil, France; ^3^Orbio, Lyon, France; ^4^Unité de Bactériologie, BioPôle, Ecole Nationale Vétérinaire d’Alfort, Maisons-Alfort, France; ^5^Laboratoire Vétérinaire Départemental, Biot, France; ^6^Unité Epidémiologie et Appui à la Surveillance, ANSES Laboratoire de Lyon – Université de Lyon, Lyon, France

**Keywords:** *E. coli*, UTI, dog, ESBL, virulence, UPEC

## Abstract

*Escherichia coli* is a ubiquitous commensal and pathogen that has also been recognized as a multi-sectoral indicator of antimicrobial resistance (AMR). Given that latter focus, such as on resistances to extended-spectrum cephalosporins (ESC) and carbapenems, the reported population structure of *E. coli* is generally biased toward resistant isolates, with sequence type (ST)131 being widely reported in humans, and ST410 and ST648 being reported in animals. In this study, we characterized 618 non-duplicate *E. coli* isolates collected throughout France independently of their resistance phenotype. The B2 phylogroup was over-represented (79.6%) and positively associated with the presence of numerous virulence factors (VFs), including those defining the extra-intestinal pathogenic *E. coli* isolates (presence of ≥2 VFs: *papA*, *sfaS, focG*, *afaD*, *iutA*, and *kpsMTII*) and those more specifically related to uropathogenic *E. coli* (*cnf1*, *hlyD*). The major STs associated with clinical isolates from dogs were by far the dog-associated ST372 (20.7%) and ST73 (20.1%), a lineage that had commonly been considered until now as human-associated. Resistance to ESC was found in 33 isolates (5.3%), along with one carbapenemase-producing isolate, and was mostly restricted to non-B2 isolates. In conclusion, the presence of virulent *E. coli* lineages may be the issue, rather than the presence of ESC-resistant isolates, and the risk of transmission of such virulent isolates to humans needs to be further studied.

## Introduction

*Escherichia coli* is both a commensal microorganism and a major pathogen in humans and animals. *E. coli* has also been recognized as a multi-sectoral indicator of the antimicrobial resistance (AMR) burden due to its large distribution in a wide range of ecosystems. Concerning AMR, most data on *E. coli* have focused on resistance to extended-spectrum cephalosporins (ESCs), conferred either by extended-spectrum beta-lactamases (ESBLs) or plasmid-mediated cephalosporinases (pAmpCs), and numerous cross-sectional studies have investigated ESC-resistant (ESC-R) *E. coli* to decipher possible transfers across sectors. In particular, several studies have demonstrated the transmission of ESC-R *E. coli* between humans and dogs, mostly within households (Ljungquist et al., 2016; Gronthal et al., 2018). Overall, the distribution of ESC-R *E. coli* in dogs shows a wide diversity of lineages together with a few dominant clones, namely of sequence type (ST)648, ST410, and to a lesser extent, the human-associated ST131 (Ewers et al., 2014; Falgenhauer et al., 2016; Melo et al., 2019).

More globally, humans and dogs share common diseases caused by extra-intestinal pathogenic *E. coli* (ExPEC) (LeCuyer et al., 2018; Manges et al., 2019), of which urinary tract infections (UTIs) are by far the most common. Transmission of ExPEC between dogs and their owners has also been described (Damborg et al., 2009; Reeves et al., 2011; Damborg et al., 2018), and contact with dogs or dog feces is considered a risk factor for the acquisition of resistant ExPEC (Ukah et al., 2018). Nonetheless, due to frequent focus on AMR, the population structure of pathogenic *E. coli* that have not been studied for their resistance phenotype remains largely under-reported. A recent study showed the dominance of specific ExPEC lineages in dogs in the United States, such as the primarily dog-associated ST372 or the human-associated ST73, ST12, or ST127 (LeCuyer et al., 2018). Uropathogenic *E. coli* (UPEC), which are a sub-pathotype of ExPEC mostly found in human infections, have often been associated with four major ST complexes: ST14, ST69, ST73, and ST95 (Lau et al., 2008; Liu et al., 2015; Fibke et al., 2019). ST73 in particular may be an underestimated human UPEC because it is not often associated with ESC-R determinants (Manges et al., 2019). Furthermore, ESC-R *E. coli* show lower virulence scores, except for the *iutA* gene (LeCuyer et al., 2018; Zogg et al., 2018b). Interestingly, a reduced virulence load in multidrug-resistant *E. coli*, concomitant to an increased prevalence of virulence genes in susceptible *E. coli* belonging to the phylogroup B2, has also been reported in isolates from Germany and Switzerland (Wagner et al., 2014; Zogg et al., 2018b).

To characterize the *E. coli* lineages causing diseases in French dogs and to assess to what extent this population structure may include ExPEC and/or UPEC lineages causing diseases in humans, we investigated 618 non-duplicate clinical *E. coli* isolates collected from dogs throughout France in 2017. This was done independently of their resistance phenotype to correctly estimate the resistance and virulence load which can potentially be transmitted to (or from) humans.

## Materials and Methods

### Bacterial Isolates

*Escherichia coli* isolates (*n* = 618) were collected from four veterinary diagnostic laboratories in France between January and November 2017. The four laboratories were asked to provide all non-duplicate *E. coli* isolates causing diseases in dogs over a continuous 3-month period of time in 2017. Two laboratories were located in the Parisian suburbs, one in the Rhône département and one in the Alpes-Maritimes département. Three laboratories were referee laboratories (the two Parisian laboratories and the one from the Rhône département), receiving samples from all across France, such that the sampling of this study was considered representative of clinical *E. coli* isolates circulating in dogs in France. Most *E. coli* isolates were recovered from UTIs (*n* = 403, 65.2%) (Supplementary Table S1), but also unexpectedly in a few cases of diarrhea (*n* = 29, 4.7%). Considering the ubiquitous presence of *E. coli*, we cannot completely exclude that a few isolates included in this study were not pathogenic. However, maximum precautions were taken to avoid this bias, since samples were all taken by veterinarians suspecting infectious diseases and since polycontaminated samples (≥3 bacteria growing on one plate) were not processed. Bacterial isolation was performed on Eosin Methylene Blue agar (EMB; ThermoFisher Diagnostics, Dardilly, France), Brillance^TM^ UTI agar (Oxoid), chromID^®^ CPS^®^ Elite agar (BioMérieux, Marcy l’Etoile, France) or blood agar, and identification was performed using MALDI-TOF mass spectrometry or API^®^ gallery-based identification according to each veterinary laboratory’s methodology. All isolates were sent to the ANSES laboratory in Lyon for further characterization.

### Antimicrobial Susceptibility Testing

Susceptibility testing was performed using the disk-diffusion test on Mueller–Hinton agar (BioRad, Marne-la-Coquette, France), according to the guidelines of the Antibiogram Committee of the French Society for Microbiology (CA-SFM, 2019) and using clinical breakpoints referenced in the veterinary part of the CA-SFM (2019). For antibiotics that are not veterinary-licensed, such as carbapenems, breakpoints recorded in the human part of the CA-SFM were used. The following antibiotics of human and/or veterinary interest were tested: amoxicillin, amoxicillin + clavulanic acid, cefalotin, cefuroxime, cefotaxime, ceftiofur, piperacillin, ticarcillin, piperacillin + tazobactam, ticarcillin + clavulanic acid, ertapenem, ceftazidime, cefoxitin, cefepime, aztreonam, cefquinome, streptomycin, kanamycin, amikacin, apramycin, gentamicin, tobramycin, netilmicin, chloramphenicol, florfenicol, tetracycline, colistin, sulfonamides, trimethoprim, nalidixic acid, enrofloxacin, and ofloxacin. *E. coli* ATCC 25922 was used as a quality control.

### Identification of β-Lactamase and Virulence Genes

Each mix PCR (20 μL) contained 2 μL of DNA lysate, primers (0.5 μM); dNTP (0.2 mM), 1 U of *Taq* polymerase (Promega GoTaq^®^ G2) and 4 μL 5X *Taq* Buffer (Promega) qsp H_2_O. Multiplex PCRs were performed using the Multiplex PCR kit (Qiagen, France) according the manufacturer’s instructions. PCR products were run and analyzed using a QIAxcel Advanced system (Qiagen, Courtaboeuf, France).

#### Resistance Genes

PCRs were performed using specific primers for the detection of the ESBL genes *bla*_CTX–M_ group 1 and group 9, and *bla*_CTX–M–__2_ (Shibata et al., 2006; Dierikx et al., 2010). For all *bla*_CTX–M_ group 1 and group 9 isolates, additional PCRs were performed to identify the exact *bla*_CTX–M_ gene variant using the ISEcp1L1/P2D and MA1/MA2 primers, respectively. The *bla*_CMY_ genes were detected using CF1/CF2 primers (Eckert et al., 2004). All positive amplicons were sequenced (Genewiz, London, United Kingdom).

#### Virulence Genes

Two multiplex PCRs targeting seven genes (*focG*, *kpsMTII*, *papA*, *sfaS*, *afaD, hlyD*, *iutA*) related to ExPEC status were performed as previously described (Franz et al., 2015). In this study only the *papA* gene from the *pap* locus was considered for the ExPEC definition. The detection of two UPEC-associated genes (*cnf1/cnf2*, and *hlyD*) was done using previously described primers (Toth et al., 2003; Johnson et al., 2010). PCRs targeting six additional ExPEC-associated virulence genes (*cdtB, iroN, ideA, iss*, *fimH*, and *fyuA)* were performed using specific primers (Peigne et al., 2009; Johnson et al., 2010; Spurbeck et al., 2012; Weissman et al., 2012). The *aggR* gene related to entero-aggregative *E. coli* (EAEC) was detected as described in a previous study (Fujioka et al., 2009). The *stx1*, *stx2*, and *eae* genes associated with the Entero-Haemorrhagic *E. coli* (EHEC) pathovar were identified using published primers (Cebula et al., 1995; Paton et al., 1995; Clermont et al., 2011).

Isolates carrying at least two of the following genes *papA*, *sfaS*-*focG*, *afaD, kpsMTII* and *iutA* were classified as ExPEC as described (Johnson et al., 2003). *E. coli* isolates that possessed at least two ExPEC determinants and the additional haemolysin secretion protein D gene *hlyD* and the cytotoxic necrotizing factor *cnf1* were classified as UPEC, as previously defined (Johnson, 2003).

### Genetic and Molecular Typing of the Strains

Phylogenetic grouping of the *E. coli* isolates was performed using a published improved method (Clermont et al., 2013). The B2-O25b-ST131 clone of *E. coli* was detected using a published PCR-based assay (Clermont et al., 2009). Multi-locus sequence typing (MLST) of all ESBL/pAmpC/carbapenem-producing isolates (*n* = 34) was performed according to the Achtman protocol described on the *E. coli* MLST website^1^. The complete MLST scheme was also performed on a randomly selected subset of 89 non-ESBL isolates belonging to the B2 phylogroup. For the remaining *E. coli* isolates belonging to the B2 phylogroup (*n* = 392), the *fumC* allele was sequenced as a reliable indicator of the MLST types ST372 (*fumC103*), ST73 (*fumC24*), ST141 (*fumC52*), and ST131 (*fumC40*). The *purA* and *icd* alleles were further sequenced for a subset of isolates presumably belonging to ST12 (*fumC13* and *purA10*), ST127 (*fumC14* and *icd36*), or ST961 (*fumC13* and *purA126*). ST131 clones were screened using PCR (Clermont et al., 2009). For ST131-positive isolates, allelic variants of *fimH* were identified by PCR and sequencing. The presence of a C1-M27 cluster was determined using previously published PCRs targeting the cluster-specific prophage-like region (M27PP1) (Birgy et al., 2017).

### Plasmid Characterization

Plasmids were typed using PCR-based replicon typing (PBRT) according to the published PBRT kit scheme using a commercial kit (Diatheva, Cartoceto, Italy) (Carattoli et al., 2005). Plasmids carrying the ESBL/pAmpC/carbapenme-resistance genes were detected using PFGE-S1 gels (6 V/cm for 20 h with an angle of 120° at 14°C with pulse times ranging from 1 to 30 s) followed by Southern blot using adequate probes (Dierikx et al., 2010). After electrical transfer of DNA to nylon membranes, Southern blots were performed according to the manufacturer’s protocol (Roche Diagnostics, Meylan, Germany). After overnight hybridization at 37°C, all membranes were treated with maleic acid and blocking solution for 45 min, then with purified anti-digoxigenin-AP Fab fragments, washing buffer and finally stained with NBT/BCIP. Plasmid co-localization was assessed by comparison between the bands corresponding to the resistance gene and those corresponding to the Inc type of the plasmid. The chromosomal location was identified by PFGE on *I-Ceu*1-digested DNA, followed by Southern blot hybridization using a 16S rDNA probe and probes specific to resistance genes.

### Whole Genome Sequencing

Whole genome sequencing (WGS) of 14 isolates was performed. Isolates were chosen because (i) they were classified as UPECs, (ii) they belonged to major STs, and (iii) they presented diverse virulence patterns. These 14 *E. coli* belonged to various STs (ST73, *n* = 4; ST372, *n* = 2; ST131, *n* = 3; ST141, *n* = 2; ST12 variant, *n* = 1; ST1262 variant, *n* = 1; ST2015, *n* = 1) was performed using the NextSeq technology (Illumina) from genomic DNA extracted from an overnight culture using the NucleoSpin^®^ Microbial DNA kit (Macherey Nagel, Germany). After trimming (using Trimmomatic), SPAdes (version 3.9) *de novo* assembly was performed using the Center for Genomic Epidemiology (CGE) server^2^. Contigs shorter than 200 bp were filtered out. Contigs were annotated using the National Center for Biotechnology Information (NCBI) Prokaryotic Genomes Automatic Annotation Pipeline (PGAP) on http://www.ncbi.nlm.nih.gov/genomes/static/Pipeline.html. Virulence factors (VFs), resistance genes, STs and serotypes were identified from the assembled genomes according to VirFinder, ResFinder, and MLST finder from CGE (see text footnote 2).

### Statistical Analysis

Fisher’s exact test was used to determine whether proportions for one variable (antimicrobial susceptibility (Table 1) or multidrug-resistance (Table 2) or the distribution of virulence gene (Table 3) are different between phylogroups. The tests were considered significant when *p*-values were ≤0.05. When the Fisher’s exact test was significant, an analysis and graphic visualization of the proportions and their 95% confidence intervals (CI) were performed. A proportion was considered significant when its CI did not overlap at least one other CI.

**TABLE 1:** Antimicrobial resistance detected in the 618 studied *Escherichia coli* isolates.

**Antibiotic**	**Number (%) of isolates**				**Total**	***p-*value (Fisher’s exact test)**
	**Phylogroup A**	**Phylogroup B1**	**Phylogroup B2**	**Phylogroup D**		
	**35 (5.7)**	**53 (8.6)**	**492 (79.6)**	**38 (6.1)**	**618 (100)**	
Amoxicillin	22 (62.9)	30 (56.6)	**92 (18.7)^1^**	26 (68.4)	170 (27.5)	<2.2E-16
AMC	12 (34.3)	10 (18.9)	**12 (2.4)**	11 (28.9)	45 (7.3)	<2.2E-16
Cefalotin	12 (34.3)	9 (17.0)	**15 (3.0)**	11 (28.9)	47 (7.6)	1.27*E*−13
Cefuroxime	18 (51.4)	16 (30.2)	**45 (9.1)**	16 (42.1)	95 (15.4)	1.652*E*−14
Cefotaxime	10 (28.6)	5 (9.4)	**12 (2.4)**	10 (26.3)	37 (6.0)	2.57*E*−11
Ceftiofur	10 (28.6)	4 (7.5)	**11 (2.2)**	9 (23.7)	34 (5.5)	8.35*E*−11
Ertapenem	0 (0)	0 (0)	0 (0)	1 (2.6)	0 (0)	>0.05
Ceftazidime	9 (25.7)	6 (11.3)	**11 (2.2)**	10 (26.3)	36 (5.8)	2.98*E*−11
Cefoxitin	1 (2.9)	7 (13.2)	**10 (2.0)**	9 (23.7)	27 (4.4)	1.08*E*−07
Cefquinome	9 (25.7)	8 (15.1)	**3 (0.6)**	3 (7.9)	23 (3.7)	1.49*E*−12
Streptomycin	15 (42.9)	18 (34.0)	**55 (11.2)**	15 (39.5)	103 (16.7)	1.72*E*−10
Kanamycin	9 (25.7)	5 (9.4)	**14 (2.8)**	7 (18.4)	35 (5.7)	7.11*E*−08
Amikacin	0 (0)	0 (0)	0 (0)	0 (0)	0 (0)	>0.05
Apramycin	0 (0)	0 (0)	0 (0)	0 (0)	0 (0)	>0.05
Gentamicin	9 (25.7)	0 (0)	**3 (0.6)**	5 (13.2)	17 (2.8)	4.29*E*−11
Tobramycin	9 (25.7)	0 (0)	**4 (0.8)**	5 (13.2)	18 (2.9)	1.56*E*−10
Netilmicin	7 (20.0)	0 (0)	**1 (0.2)**	1 (2.6)	9 (1.5)	3.0*E*−8
Chloramphenicol	13 (37.1)	3 (5.7)	**23 (4.7)**	6 (15.8)	45 (7.3)	4.53*E*−08
Tetracycline	20 (57.1)	12 (22.6)	**48 (9.8)**	21 (55.3)	101 (16.3)	<2.2E-16
Colistin	0 (0)	0 (0)	0 (0)	0 (0)	0 (0)	>0.05
Sulfonamides	18 (51.4)	20 (37.7)	**72 (14.6)**	18 (47.4)	128 (20.7)	2.84*E*−11
Trimethoprim	14 (40.0)	19 (35.8)	**28 (5.7)**	16 (42.1)	77 (12.5)	<2.2E-16
Nalidixic acid	14 (40.0)	14 (26.4)	**29 (5.9)**	12 (31.6)	69 (11.2)	6.22*E*−13
Enrofloxacin	12 (34.3)	10 (18.9)	**5 (1.0)**	9 (23.7)	36 (5.8)	<2.2E-16

*AMC, amoxicillin + clavulanic acid. ^1^Numbers indicate *p-*values (Fisher’s exact test). Numbers in bold indicate statistically significant values. For each antibiotic, the Fisher’s exact test was used to compare the proportions of resistant isolates among phylogroups. When the Fisher’s exact test was significant, an analysis and graphic visualization of the proportions and their 95% confidence intervals (CI) were performed. A proportion was considered significant when its CI did not overlap at least one other CI.*

**TABLE 2:** Multidrug-resistance according to phylogroup.

**Phylogroup**	**Total (*n*)**	**No. of isolates resistant to 0–7 antibiotic families^1^**								**No. of MDR**	**% of MDR**	***p-*value (Fisher’s exact test)**
		**0**	**1**	**2**	**3**	**4**	**5**	**6**	**7**			
A	35	11	1	7	1	4	1	3	7	16	45.7	
B1	53	21	6	6	7	9	4	0	0	20	37.7	
B2	492	377	44	27	23	14	7	0	0	44	8.9	
D	38	11	3	5	4	7	3	1	4	19	50.0	3.99E-17
Total	618	420	54	45	35	34	15	4	11	99	16.0	

*^1^Antibiotics considered for determination of multidrug-resistance: amoxicillin, ceftiofur, streptomycin, chloramphenicol, tetracyclines, sulfonamides-trimethoprim, and enrofloxacin. MDR, multidrug-resistance. Isolates considered as non-multidrug-resistant are those resistant to 0–2 antibiotic families, while isolates considered as multidrug-resistant are those resistant to 3–7 antibiotic families. The Fisher’s exact test was used to compare the proportion of multidrug-resistant (MDR) isolates among phylogroups, *p-*value was calculated from the number of MDR isolates for each phylogroups.*

**TABLE 3:** Distribution of virulence-associated genes among the A, B1, B2, and D phylogroups (618 studied *Escherichia coli* isolates).

**Virulence gene**	**Number (%) of isolates**				**Total**	***p-*value^2^**
	**Phylogroup A**	**Phylogroup B1**	**Phylogroup B2**	**Phylogroup D**		
	**35 (5.7)**	**53 (8.6)**	**492 (79.6)**	**38 (6.1)**	**618 (100)**	
*papA*	13 (37.1)	6 (11.3)	**306 (62.2)^1^**	4 (10.5)	329 (53.2)	<2.2E-16
*kpsMTII*	4 (11.4)	2 (3.8)	307 (62.4)	**24 (63.2)**	337 (54.5)	1.31E-23
*sfaS*	0 (0.0)	0 (0.0)	**79 (16.1)**	0 (0.0)	79 (12.8)	1.19E-06
*afaD*	0 (0.0)	0 (0.0)	1 (0.2)	1 (2.6)	2 (0.3)	0.23
*iutA*	19 (54.3)	20 (37.7)	**48 (9.8)**	17 (44.7)	104 (16.8)	<2.2E-16
*fyuA*	16 (45.7)	22 (41.5)	**402 (81.7)**	19 (50.0)	459 (74.3)	3.08E14
*focG*	0 (0.0)	2 (3.8)	**193 (39.2)**	1 (2.6)	196 (31.7)	<2.2E-16
*hlyD*	7 (20.0)	3 (5.7)	**313 (63.6)**	3 (7.9)	326 (52.8)	<2.2E-16
*cnf1*	7 (20.0)	2 (3.8)	**315 (64.0)**	2 (5.3)	326 (52.8)	<2.2E-16
*cdt3*	0 (0.0)	1 (1.9)	0 (0.0)	0 (0.0)	1 (0.2)	1
*fimH*	32 (91.4)	47 (88.7)	422 (85.8)	32 (84.2)	533 (86.2)	0.78
*iroN*	17 (48.6)	22 (41.5)	294 (59.8)	21 (55.3)	354 (57.3)	0.051
*ibeA*	6 (17.1)	7 (13.2)	**162 (32.9)**	4 (10.5)	179 (29.0)	1.65E-4
*iss*	9 (25.7)	16 (30.2)	**59 (12.0)**	12 (31.6)	96 (15.5)	4.56E-05
*eae*	2 (5.7)	1 (1.9)	5 (1.0)	0 (0.0)	8 (1.3)	0.113
Median VS^2^	3	3	6	4	6	–
IQ min	1	0	0	0	0	–
IQ max	9	8	10	8	10	–
ExPEC	14 (40.0)	7 (13.2)	**333 (67.7)**	15 (39.5)	369 (59.7)	2.54E-16
UPEC	7 (20.0)	2 (3.8)	**263 (53.5)**	2 (5.3)	274 (44.3)	9.41E-22

*^1^Numbers indicate p-values (Fisher’s exact test). Numbers in bold indicate statistically significant values. ^2^*p*-value (Fisher’s exact test) calculated from the number of positive isolates for each virulence gene among phylogroups. When the Fisher’s exact test was significant, an analysis and graphic visualization of the proportions and their 95% confidence intervals (CI) was performed. A proportion was considered significant when its CI did not overlap at least with one other CI. ^2^VS is the Median virulence-associated gene Score calculated as the sum of virulence genes found positive using PCR. IQ: interquartile range of the virulence gene scores. ExPEC status was defined as the presence of ≥2 of the following genes: *papA, focG, kpsMTII*, *sfaS*, *afaD*, and *iutA.* UPEC status was defined as the presence of the *hlyD* and *cnf1* genes among ExPEC isolates. The virulence genes *cnf2*, *stx1*, *stx2*, *aggR*, and *cdt3* were absent from this population of isolates.*

Univariate logistic regression model was implemented to assess the associations between ESBL or AmpC or pathology (here UTI) status and the presence of virulence genes: the ESBL (or AmpC or pathology) status was the dependent variable and each model included a bimodal explanatory variable related to the studied virulence gene regardless of the presence (or not) of other genes. The *p*-values, odds ratio (OR) and 95% confidence intervals were calculated by a logistic regression model univariate (Table 4). A multivariate model (taking into account the 12 virulence genes detected in this study as well as their interactions) could not be implemented since there were too many parameters to estimate in relation to the available data and the model would not converge. Associations were considered significant at *p* ≤ 0.05. R software version 3.6.0 (2019-04-26) was used for statistical analyses (R Core Team, 2019).

**TABLE 4:** Association between ESBL, AmpC or UTI status, and virulence genes.

**Status**	**Virulence gene**	***p*-value**	**Odd Ratio**	**95% CI**
ESBL	*iutA*	4E-05	73.29	14.34–1,339.49
	*iroN*	0.03838	0.29	0.08–0.88
AmpC	*kpsMII*	0.00156	0.09	0.01–0.33
UTIs	*cnf1*	0.02353	1.47	1.05–2.05
	*focG*	0.0061	1.68	1.17–2.45
	*papA*	0.00104	1.75	1.25–2.45
	*sfaS*	0.03376	1.8	1.07–3.19
	*hlyD*	0.01498	1.51	1.08–2.11

*The *p*-values, odds ratio and 95% confidence intervals (CI) were determined by univariate logistic regression.*

## Results

### Distribution of Phylogroups and Lineages of Pathogenic *E. coli* From Dogs

**FIGURE 1:**
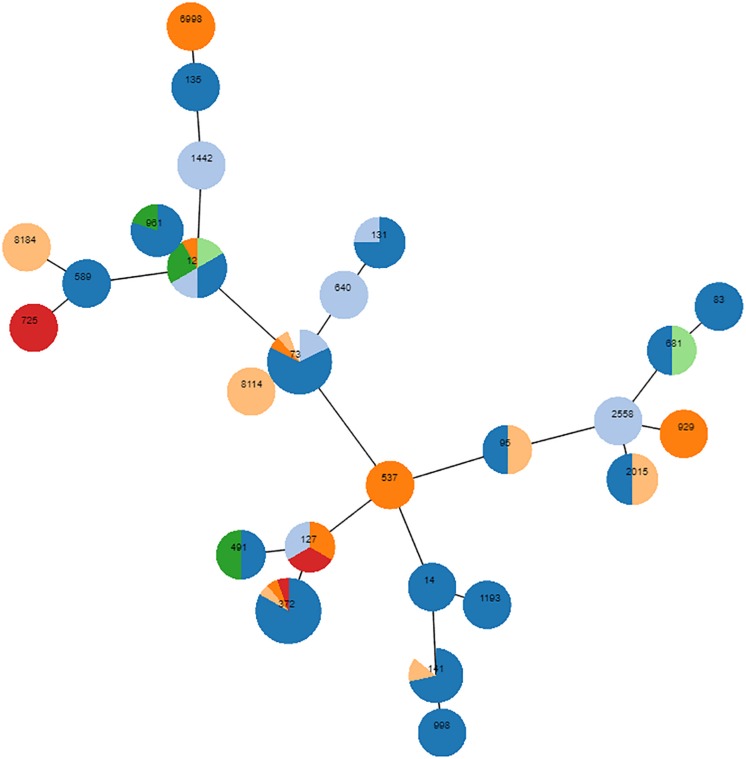
Phylogenetic proximity of 89 randomly chosen B2 isolates, as inferred using goeBURST (phyloviz). Each circle is a pie chart showing the pathologies from which the isolates were obtained. Digestive disease is colored in red, UTIs in dark blue, unknown pathology in light blue, skin infection in orange, otitis in light orange, reproduction disorder in green, respiratory disease in light green. The node size of the goeBURST is proportional to the number of isolates. Nodes are in log scale. Scaling factor = 2.

A total of 618 *E. coli* isolates were collected, of which 35 belonged to phylogroup A (5.7%), 53 to B1 (8.6%), 492 to B2 (79.6%) and 38 to phylogroup D (6.1%). Most isolates (65.2%) were associated with UTIs, but several other diseases were diagnosed (Supplementary Table S1). In all diseases, B2 was by far the predominant phylogroup, ranging from 58.6% (*n* = 17, digestive tract infections) to 90% (*n* = 54, otitis) of the isolates (Supplementary Table S1). According to this over-representation of the B2 phylogroup, complete MLST of a randomly chosen subset of 89 B2 *E. coli* revealed dominant STs, with ST372 (*n* = 16, 17.9%) and ST73 (*n* = 16, 17.9%) being the most frequent (Figure 1). ST12 (*n* = 9), ST141 (*n* = 7), and ST961 (*n* = 5) were also repeatedly identified, and ST131 was detected in two animals. Sixteen STs were identified once, three twice and two STs were detected three times. Finally, six STs were not found in the Achtman database. To further decipher the major lineages composing the 492 B2 *E. coli* group, partial MLST was performed: the *fumC* allele was sequenced on all 403 remaining isolates, and the *purA* and *icd* alleles were subsequently sequenced on a subset of isolates to refine the analysis. Over the total number of B2 *E. coli*, 102 belonged to ST372 (102/492, 20.7%) and 99 to ST73 (20.1%), slightly modifying the previously estimated proportions of both STs (both initially estimated at 17.9%). The other STs were found in smaller numbers, with 37 isolates belonging to ST141 (7.5%), 13 to ST12 (2.6%), 12 to ST127 (2.4%), 9 to ST131 (1.8%), and 6 to ST961 (1.2%). Among the nine ST131, eight showed an unusual *fimH* variant, and the last one showed the *fimH*30 allelic variant.

### Resistance Profiles of Pathogenic *E. coli* From Dogs

Resistances to amoxicillin (27.5%), sulfonamides (20.7%), streptomycin (16.7%) and tetracyclines (16.3%) were most frequent (Table 1). Although phylogroups A, B1, and D include fewer isolates (*n* = 126) than phylogroup B2 (*n* = 492), isolates belonging to the former three phylogroups presented statistically much more resistances than isolates from phylogroup B2. For example, enrofloxacin resistance was observed in 1% of the B2 isolates (*n* = 5), but occurred in 24.6% of the non-B2 isolates (*n* = 31). This discrepancy was also clearly demonstrated by the occurrence of multidrug-resistance (resistance to ≥3 antibiotic families), which was detected in 8.9% of the B2 and 43.7% of the non-B2 isolates (Table 2).

ESC-resistance was identified in 33 isolates (5.3%), including 14 ESBL- and 19 AmpC-producing *E. coli*. All these latter isolates produced the CMY-2 enzyme and all ESBL-producing *E. coli* produced a CTX-M-type enzyme. CTX-M-15 was the most frequent (*n* = 10), followed by CTX-M-27 (*n* = 2), CTX-M-1 (*n* = 1) and CTX-M-3 (*n* = 1). The 19 CMY-2-producing *E. coli* presented very diverse genetic backgrounds, since 13 different STs were identified. Only *E. coli* ST12 and ST4608 were each detected in three different dogs. On the contrary, ST410 was identified in 7/14 of the ESBL-producing *E. coli*. Of note, one ST131 isolate producing CTX-M-27 belonged to the C1-M27 sub-clade. A single isolate was resistant to ertapenem (0.2%) and identified as an OXA-48 producer belonging to ST1485. All *bla*_CMY–__2_ genes were carried by IncI1 plasmids. The *bla*_CTX–M–__27_ and *bla*_CTX–M–__1_ genes were located on IncF plasmids and the *bla*_OXA–__48_ was carried by an IncL plasmid. In contrast, all *bla*_CTX–M–__15_ genes were found on the bacterial chromosome.

### Virulence Profiles of Pathogenic *E. coli* From Dogs

Among the 618 isolates, 369 (59.7%) were classified as ExPEC (≥2 VFs among *papA*, *sfaS, focG*, *afaD*, *iutA*, *kpsMTII*). Most ExPEC isolates belonged to phylogroup B2 (*n* = 333/369, 90.2%). The proportion of ExPEC isolates from phylogroup B2 was 67.7% (*n* = 333/492) and significantly lower in phylogroup B1 (*n* = 7/53, *p* = 2.54E-16) (Table 3). A total of 274 ExPEC were sub-pathotyped as UPEC (ExPEC + *hlyD* and *cnf1*). Among the 274 UPEC, 196 (71.5%) isolates were associated with UTIs, 24 (8.8%) with otitis, 13 (4.7%) with reproduction disorder, 8 (2.9%) with skin infection, 8 (2.9%) with digestive disease, 7 (2.5%) with respiratory diseases and 18 (6.6%) were from unknown pathology. The significance of UPEC isolates among digestive tract infections can be questioned. Given the low number of isolates (*n* = 8), this may be due to the transitory presence of a B2 isolate in the gastrointestinal tract. UPEC isolates belonged to B2 for the most part (*n* = 263/274, 96.0%, *p* = 1.53E-23). The proportion of UPEC among B2 isolates reached 53.5% (*n* = 263/492) (Table 3) and 43% of UPEC belonged to ST372 (*n* = 59/105) or ST73 (*n* = 56/99).

Virulence gene scores varied from 0 to 10 among phylogroups. The median virulence gene score was higher in phylogroup B2 than in the A, B1, and D phylogroups (Table 3). However, the distribution of virulence-associated genes varied among phylogroups (Supplementary Figure S1 and Table 3). The siderophore-encoding *fyuA* gene was the most frequently identified virulence factor (81.7%) in the B2 group. This *fyuA* gene, along with *papA* (adhesin, 62.2%), *kpsMTII* (protectin, 62.4%), *sfaS* (adhesin, 16.1%), *focG* (adhesin, 39.2%), *hlyD* (toxin, 63.6%), *cnf1* (toxin, 64%) and *ibeA* (invasin, 32.9%), was highly associated with phylogroup B2 (Table 3). On the contrary, the proportions of the *iutA* (siderophore, 9.8%) and *iss* (protectin, 12.0%) genes were significantly lower in B2 isolates (Table 3). The *papA* (*p* = 0.053), *fyuA* (*p* = 4.3E-5), *focG* (6.44E-11), *hlyD* (*p* = 0.007) and *cnf1* (0.013) VFs were related to ST372 compared to the other isolates using the Fisher’s exact test. *focG* (*p* = 3.21E-4), *hlyD* (*p* = 0.011) and *cnf1* (*p* = 0.006) were also linked to ST73. The *iutA* (*p* = 0.021), *kpsMTII* (*p* = 0.031) and *iss* (*p* = 1.4E-4) genes were rarely found in ST372. The virulence profiles among UTI-associated ESBLs and AmpC were described in Table 5 and Supplementary Table S3.

**TABLE 5:** Molecular characteristics of the 34 extended-spectrum cephalosporin- and carbapenem-resistant isolates.

**Strain**	**Pathology**	**Sequence type**	**Phylogroup**	**Phenotype**	**Beta-lactamase enzyme**	**Associated resistances**	**Virulence profiles**
A14	UTI	191	A	AmpC	CMY-2	None	*fimH*
A08	UTI	NT	B2	AmpC	CMY-2	CHL, TET, SUL, TMP, NAL	*fimH, iutA, iroN*
B03	UTI	4,608	D	AmpC	CMY-2	STR, KAN, GEN, CHL, TET, SUL, TMP, NAL, ENR	*fimH, cnf1, papA, hlyD, iutA, iroN, iss, fuyA*
C05	Unknown	12	B2	AmpC	CMY-2	None	*fimH, cnf1, focG, papA, hlyD, iroN, ibeA, fuyA*
D08	UTI	372	B2	AmpC	CMY-2	None	*fimH, focG, ibeA, fuyA*
D09	Reproductive tract	1,706	B1	AmpC	CMY-2	None	*fimH*
E03	UTI	297	B1	AmpC	CMY-2	STR, SUL, TMP	*fimH*
F06	UTI	4,608	D	AmpC	CMY-2	STR, KAN, GEN, CHL, TET, SUL, TMP, NAL, ENR	*papA, iutA, iss*
F10	UTI	12	B2	AmpC	CMY-2	STR, KAN, CHL, SUL, NAL	*fimH, cnf1, papA, hlyD, iroN, fuyA*
I54	Unknown	141	B2	AmpC	CMY-2	STR, CHL, TET, SUL	*fimH, cnf1, papA, hlyD, iroN, fuyA*
I58	Otitis	162	B1	AmpC	CMY-2	STR, TET, SUL, NAL, ENR	*fim, fuyA*
K26	UTI	212	B1	AmpC	CMY-2	None	*fimH*
N19	Digestive	NT	B2	AmpC	CMY-2	None	*focG, fimH, fuyA*
Q05	UTI	4,608	D	AmpC	CMY-2	STR, KAN, GEN, CHL, TET, SUL, TMP, NAL, ENR	*fimH, cnf1, papA, hlyD, iutA, iroN, fuyA*
Q29	Digestive	1,011	D	AmpC	CMY-2	STR, TET, SUL, TMP, NAL, ENR	*fimH, iroN, ibeA*
R07	UTI	2,015	B2	AmpC	CMY-2	None	*fimH, cnf1, kpsMII, papA, iroN, ibeA, fuyA*
T11	UTI	68	D	AmpC	CMY-2	NAL	*kpsMII, fimH, fuyA*
X25	UTI	372	B2	AmpC	CMY-2	None	*fimH, cnf1, focG, papA, sfaS, hlyD, fuyA*
Z01	Respiratory	12	B2	AmpC	CMY-2	None	*fimH, cnf1, focG, papA, hlyD, iroN, fuyA*
T12	Respiratory	1,485	D	Carbapenemase	OXA-48	STR, TET, SUL, TMP	*kpsMII, fimH*
B04	UTI	410	A	ESBL	**CTX-M-15^3^**	STR, KAN, GEN, CHL, TET, SUL, TMP, NAL, ENR	*fimH, cnf1, papA, hlyD, iutA, fuyA*
B11	UTI	648	D	ESBL	CTX-M-3	STR, KAN, GEN, CHL, TET, SUL, TMP, NAL, ENR	*fimH, kpsMII, iutA*
C17	UTI	131	B2	ESBL	CTX-M-27	NAL, ENR	*fimH, kpsMII, iutA*
E08	UTI	410	A	ESBL	**CTX-M-15**	STR, KAN, GEN, CHL, TET, SUL, TMP, NAL, ENR	*papA, iutA*
H20	UTI	90	A	ESBL	CTX-M-27	STR, TET, SUL, TMP, NAL, ENR	*fimH*
I47	UTI	372	B2	ESBL	**CTX-M-15**	CHL	*kpsMII, iutA, ibeA, fuyA*
N01	UTI	410	A	ESBL	**CTX-M-15**	STR, KAN, GEN, CHL, TET, SUL, TMP, NAL, ENR	*fimH, cnf1, papA, hlyD, iutA, ibeA, fuyA*
N11	UTI	410	A	ESBL	**CTX-M-15**	STR, KAN, GEN, CHL, TET, SUL, TMP, NAL, ENR	*fimH, cnf1, papA, hlyD, iutA, fuyA*
N20	UTI	410	A	ESBL	**CTX-M-15**	STR, KAN, GEN, CHL, TET, SUL, NAL, ENR	*cnf1, papA, hlyD, iutA, fuyA*
P24	UTI	23	A	ESBL	CTX-M-1	TET, SUL, TMP	*fimH, iutA, iroN, ibeA*
T01	UTI	410	A	ESBL	**CTX-M-15**	STR, KAN, GEN, CHL, TET, SUL, TMP, NAL, ENR	*fimH, cnf1, papA, hlyD, iutA, iroN, fuyA*
T03	Reproductive tract	NT	A	ESBL	**CTX-M-15**	STR, KAN, GEN, CHL, TET, SUL, TMP, NAL, ENR	*fimH, cnf1, kpsMII, papA, hlyD, iutA, iroN, ibeA, fuyA*
T13	UTI	648	D	ESBL	**CTX-M-15**	KAN, TET, NAL, ENR	*kpsMII, papA, afa, hlyD, iutA, iroN, ibeA, fuyA*
Z09	UTI	410	A	ESBL	**CTX-M-15**	STR, KAN, GEN, CHL, TET, SUL, TMP, NAL, ENR	*fimH, cnf1, papA, hlyD, iutA, fuyA*

*UTI: urinary tract infection. ^1^Antibiotics: STR, streptomycin; KAN, kanamycin; GEN, gentamicin; CHL, chloramphenicol; TET, tetracycline; SUL, sulfonamides; TMP, trimethoprim; NAL, nalidixic acid, ENR, enrofloxacin. ^2^The A08 isolate is a *fumC* variant of ST58; N19 is a *gyrB* variant of ST372; T03 is a *purA* variant of ST410. ^3^In bold are all isolates that are chromosomally encoded as shown by Southern blots on I-*Ceu*I PFGE.*

The univariate logistic regression model showed that *iutA* was most frequently present in ESBL-producing isolates, but the *iroN* and *kpsMTII* genes were less frequently present in ESBL- or AmpC-positive isolates. The *cnf1*, *focG*, *papA*, *sfaS*, and *hlyD* genes mostly found in B2 were also associated with UTIs (Table 4).

### Whole Genome Sequencing of Pathogenic *E. coli* From Dogs

WGS was further performed on a subset of 14 UPEC isolates representative of the main STs found in dogs. Additional VFs such as *gad* (glutamate decarboxylase), *mcmA* (methylmalonyl-CoA mutase), *mch* operon involved in biosynthesis, *pic* (serine protease autotransporter), *vat* (vacuolating autotransporter toxin), *ireA* (iron-regulated outer membrane) and *ihA* (iron-regulated gene homologue adhesion) were detected in all UPEC (Supplementary Table S2). The serotypes of these UPEC were respectively O15:H31 (*n* = 1), O2:H1 (*n* = 1), O2:H14 (*n* = 1), O2:H6 (*n* = 1), O25:H1, O25:H4, O4:H31 (*n* = 1), O4:H5 (*n* = 1), O6/O4:H1/H5 (*n* = 1), O6:H1 (*n* = 2) (Supplementary Table S2). Apart from the *mdf*(A) gene which was present in all isolates, only four isolates presented additional resistance genes (Supplementary Table S2). The phenotypes observed by disc diffusion for these four isolates totally matched with the detected genes.

## Discussion

In this study, we showed that the population structure of *E. coli* isolated from diseased dogs was clearly dominated by the B2 phylogroup, which accounted for 79.6% of all *E. coli* isolates. The predominance of the B2 phylogroup was most likely associated with the study design. Other studies on diseased animals with no AMR criteria have also reported high proportions of the B2 phylogroup (Liu et al., 2016; LeCuyer et al., 2018), and studies from healthy animals and/or focusing on ESC-resistant *E. coli* tended to report non-B2 phylogroups (Wagner et al., 2014). A recent exception to this pattern is a study on clinical ESC-R *E. coli* in dogs in France, which reported a high prevalence of multidrug-resistant B2 *E. coli*, but mainly linked to a high prevalence of ST131/H30 isolates (Melo et al., 2019). The low prevalence of ST131/H30 in our study confirms this clone as a human-associated pathogenic *E. coli* lineage, which occasionally transfers to companion animals.

In the present study, ST372 and ST73 were by far the dominant STs representing 20.7 and 20.1% of the B2 isolates, respectively. The seven ST372, ST73, ST141, ST12, ST127, ST131, and ST961 comprised 56.3% of all B2 isolates, which is very similar to what has been described in the United States in a study with comparable settings (LeCuyer et al., 2018). Similar findings in these two geographic areas strongly suggest a dog-specific distribution of pathogenic *E. coli* clones rather than the effect of regional factors. Of note, among the nine ST131 identified in this study, eight did not belong to the human-associated ST131 sublineages and carried unknown *fimH* variants. The last isolate produced a CTX-M-27 enzyme and belonged to the newly described C1-M27 clade. This clade first emerged in Japan before being identified in humans in other countries and continents. It has only recently been described in dogs – concomitantly in France and the United Kingdom – and this new occurrence of a C1-M27/ST131 isolate in a dog proves that this clade is not restricted to humans (Bortolami et al., 2019; Melo et al., 2019).

ST372, which has already been reported in dogs and much more sporadically in humans (Adler et al., 2012; Wagner et al., 2014), is thus emerging as a major dog-associated ST that occasionally carries resistance genes, as described here for ESBLs. Of note, ST372 was also recently reported as an OXA-48 producer (Melo et al., 2017). Its frequency in dogs most likely relies upon specific host adaptation abilities, allowing its spread in France and the United States despite the fact that the two countries differ in terms of antibiotic treatments, especially since France has set up a National Action Plan in 2012 to reduce antibiotic use (ANSES, 2018). Nonetheless, an Australian study on dogs reported the absence of ST372, which may be due to the strong border policies to avoid importation of foreign animals, but also most likely to the fact that only healthy and not clinical animals were sampled (Bourne et al., 2019). Apart from ST372, other frequent clones were human-associated STs, such as ST73, ST12, or ST127. Though usually considered a human-specific lineage, ST73 is most probably an ubiquitous urinary pathogen, because it has recurrently been described in animals, both in diseased and healthy contexts (Wagner et al., 2014; Liu et al., 2015; Bourne et al., 2019). Also, ST73 may well be under-reported in the literature because most papers have focused on ESC-R genes, which are less frequently found in this ST than in other ones such as ST131 or ST38 (Hertz et al., 2016; Manges, 2016; Van Hout et al., 2020).

The prevalence of ESC-R *E. coli* in this study was 5.3% (33/618), which is fairly low compared with what has recently been described in the United Kingdom (18.4%), Switzerland (32.1%) or even in France in 2011 (18.5%) (Zogg et al., 2018a; Bortolami et al., 2019). This low prevalence may result from the strong policy measures on AMR in the vet sector since 2012 through the National Action Plan Ecoantibio, which resulted in a 65.5% decrease in the use of last-generation cephalosporins in cats and dogs between 2013 and 2017 (ANSES, 2018). Yet, despite these efforts, carbapenem-resistant *E. coli* isolates are emerging in dogs with no apparent risk factors. ST1485 OXA-48 *E. coli* found here is the second report of an OXA-48-producing *E. coli* after the occurrence of an OXA-48 ST372 *E. coli* in a healthy dog in 2015 (Melo et al., 2017). It questions the origin of these isolates since, unlike in other countries, the use of carbapenems is totally forbidden in France, even in emergency veterinary medicine. The emergence of OXA-48 in dogs has also been demonstrated in neighboring countries such as Germany (Pulss et al., 2018), so that monitoring of carbapenem-resistance in companion animals is now strongly recommended.

Among ESC-R isolates, the fluoroquinolone-resistant ST410 clone was found in 7/14 of the ESBL-producing *E. coli*. ST410 has recently been claimed to be an international high-risk clone, similar to ST131 in humans, for its capacity to combine resistance and virulence determinants as well as to cause hospital outbreaks (Roer et al., 2018). ST410 has been reported in dogs mostly in Europe (Germany, Switzerland, United Kingdom), but also in the United States; therefore, its emergence as a major ESBL-producing pathogen in dogs should be monitored (Falgenhauer et al., 2016; Timofte et al., 2016; LeCuyer et al., 2018; Zogg et al., 2018b; Bortolami et al., 2019). In this study, our samples originated from dogs that had no epidemiological link among each other. However, all animals visited veterinary hospitals working closely with the veterinary laboratories involved, so that it may be hypothesized that this clone is circulating in veterinary hospitals. However, this remains to be proven since other sources (such as a high prevalence in the community) cannot be excluded. The *bla*_CTX–M–__15_ gene associated with ST410 was systematically carried on the bacterial chromosome, as already reported in Germany (Falgenhauer et al., 2016). Interestingly, the three remaining *bla*_CTX–M–__15_ genes found on a single-locus variant of ST410, ST372, and ST648, respectively, were also found on the chromosome. Chromosomal insertion of *bla*_CTX–M_ genes has long been considered as less frequent than their location on plasmids, but recent convergent studies suggest that it would probably be worth reconsidering this point. The factors driving chromosomal insertions are unclear. They may potentially involve bacterial fitness and certainly deserve further investigation. ST648 has also been described as a pandemic clone in animals, and has been recurrently associated either with ESBL or pAmpC enzymes (Ewers et al., 2014; Marques et al., 2018). In this study, it was identified twice as an ESBL carrier.

In agreement with previous studies, most VFs related to UPEC (*cnf1*, *focG*, *papA*, *sfaS*, and *hlyD*) were found in isolates from the B2 phylogroup. Our data confirm the low prevalence of the UPEC-associated VFs among resistant isolates, and especially in ESC-R isolates (Harwalkar et al., 2014; Hashemizadeh et al., 2017). *iutA* was the only exception, because this virulence gene was most frequently present in ESBL-producing *E. coli*, as previously suggested (LeCuyer et al., 2018). A strong correlation between the UPEC-associated VFs and the primarily dog-associated ST372 was demonstrated here. Among the VFs, *cnf1*, and *hlyD* were associated with both ST372 and ST73. Most VFs and serotypes identified in this study using the WGS analysis have been previously characterized in UPEC strains related to UTIs (Johnson and Russo, 2005; Yamamoto, 2007; Subashchandrabose and Mobley, 2015). All these results suggest a link between UPEC-associated VFs, B2 phylogroup, ST372/ST73 and UTIs in dogs.

In conclusion, to our best knowledge based on the largest pathogenic *E. coli* collection in dogs, our study showed that the population of *E. coli* in this animal species was dominated by ExPEC lineages. Although ST372 is most probably largely adapted to dogs, the human-associated ST73 appears more ubiquitous than previously thought. We also showed a clear discrepancy between resistance phenotypes and virulence-associated genes, which clustered in the non-B2 and B2 isolates, respectively. Considering that ESC-R *E. coli* only account for 5.3% of the population, but B2 isolates represent 79.6% of this same population, transmission of virulent ExPEC/UPEC lineages may well be the issue, rather than the transmission of ESC-R isolates. The risk for humans of being infected or colonized by such isolates remains to be determined. Further studies on *E. coli* populations from various hosts, irrespective of their AMR status, are surely needed to refine the scientific opinions on risks associated with inter-sectorial transfers of pathogenic *E. coli* in a “One Health” approach.

## Data Availability Statement

The whole genome shotgun project was deposited in DDBJ/EMBL/GenBank under the BioProject accession number PRJNA576337. The version described in this article is XXXX01000000. The 14 genome accession numbers are: WIKS00000000, WIKR00000000, WIKQ00000000, WIKP00000000, WIKO00000000, WIKN00000000, WIKM000- 00000, WIKL00000000, WIKK00000000, WIKJ00000000, WIKI00000000, WIKH00000000, WIKG00000000, and WIKF00000000.

## Ethics Statement

Ethical review and approval was not required for the animal study because No ethical approval was needed since this study did not involve any experimentation on animals. Only bacteria collected from clinical samples were used in the frame of this study. Written informed consent for participation was not obtained from the owners because No consent was needed since the study involved clinical samples and no metadata were recorded.

## Author Contributions

CV, MH, and J-YM designed the experiments. SB, RP, H-JB, and VB supervised the sampling campaign. AD, CV, and MH performed the experiments. CV, MH, and J-YM analyzed the data. GC and CV performed statistical analyses. MH and CV drafted the manuscript. J-YM actively contributed to the manuscript’s writing. All authors approved the final version of the manuscript.

## Conflict of Interest

SB was employed by the Vebio veterinary laboratory in Arcueil. VB was employed by the Orbio veterinary laboratory in Lyon. RP was employed by the Veterinary Department Laboratory of the Alpes-Maritimes.

The remaining authors declare that the research was conducted in the absence of any commercial or financial relationships that could be construed as a potential conflict of interest.

## Footnotes

1 https://pubmlst.org/bigsdb?db=pubmlst_mlst_seqdef

2 http://www.genomicepidemiology.org/

## References

[B1] Adler A. Gniadkowski M. Baraniak A. Izdebski R. Fiett J. Hryniewicz W. (2012). Transmission dynamics of ESBL-producing *Escherichia coli* clones in rehabilitation wards at a tertiary care centre. *Clin Microbiol Infect* 18 E497–E505. 10.1111/j.1469-0691.2012.03999.x 22963432

[B2] ANSES (2018). *Sales survey of veterinary medicinal products containing antimicrobials in France - 2017.* Buenos Aires: French Agency for Food, Environmental and Occupational Health & Safety (ANSES).

[B3] Birgy A. Bidet P. Levy C. Sobral E. Cohen R. Bonacorsi S. (2017). CTX-M-27-producing *Escherichia coli* of Sequence Type 131 and clade C1-M27. *France. Emerg Infect Dis* 23 885. 10.3201/eid2305.161865 PMC5403054 28418829

[B4] Bortolami A. Zendri F. Maciuca E. I. Wattret A. Ellis C. Schmidt V. (2019). Diversity, virulence, and clinical significance of Extended-Spectrum beta-Lactamase- and pAmpC-producing *Escherichia coli* from companion animals. *Front Microbiol* 10:1260. 10.3389/fmicb.2019.01260 31231344 PMC6560200

[B5] Bourne J. A. Chong W. L. Gordon D. M. (2019). Genetic structure, antimicrobial resistance and frequency of human associated *Escherichia coli* sequence types among faecal isolates from healthy dogs and cats living in Canberra. *Australia. PLoS One* 14 e0212867. 10.1371/journal.pone.0212867 30830915 PMC6398920

[B6] Carattoli A. Bertini A. Villa L. Falbo V. Hopkins K. L. Threlfall E. J. (2005). Identification of plasmids by PCR-based replicon typing. *J Microbiol Methods* 63 219–228. 15935499 10.1016/j.mimet.2005.03.018

[B7] CA-SFM (2019). *Comité de l’antibiogramme de la Société Française de Microbiologie: recommandations vétérinaires.* https://www.sfm-microbiologie.org/2019/07/09/casfm-veterinaire-2019/ [Online]. [Accessed]

[B8] Cebula T. A. Payne W. L. Feng P. (1995). Simultaneous identification of strains of *Escherichia coli* serotype O157:H7 and their Shiga-like toxin type by mismatch amplification mutation assay-multiplex PCR. *J Clin Microbiol* 33 248–250. 7535315 10.1128/jcm.33.1.248-250.1995 PMC227922

[B9] Clermont O. Christenson J. K. Denamur E. Gordon D. M. (2013). The Clermont *Escherichia coli* phylo-typing method revisited: improvement of specificity and detection of new phylo-groups. *Environ Microbiol Rep* 5 58–65. 10.1111/1758-2229.12019 23757131

[B10] Clermont O. Dhanji H. Upton M. Gibreel T. Fox A. Boyd D. (2009). Rapid detection of the O25b-ST131 clone of *Escherichia coli* encompassing the CTX-M-15-producing strains. *J Antimicrob Chemother* 64 274–277. 10.1093/jac/dkp194 19474064

[B11] Clermont O. Olier M. Hoede C. Diancourt L. Brisse S. Keroudean M. (2011). Animal and human pathogenic *Escherichia coli* strains share common genetic backgrounds. *Infection, Genetics and Evolution* 11 654–662. 10.1016/j.meegid.2011.02.005 21324381

[B12] Damborg P. Gumpert H. Johansson L. Jana B. Frimodt-Møller N. Guardabassi L. (2018). Dogs as reservoirs of *Escherichia coli* strains causing urinary tract infection in their owners. *bioRxiv* 302885. 10.3390/antibiotics12081269 PMC10451620 37627689

[B13] Damborg P. Nielsen S. S. Guardabassi L. (2009). *Escherichia coli* shedding patterns in humans and dogs: insights into within-household transmission of phylotypes associated with urinary tract infections. *Epidemiol Infect* 137 1457–1464. 10.1017/S095026880900226X 19272200

[B14] Dierikx C. Van Essen-Zandbergen A. Veldman K. Smith H. Mevius D. (2010). Increased detection of extended spectrum beta-lactamase producing *Salmonella enterica* and *Escherichia coli* isolates from poultry. *Vet Microbiol* 145 273–278. 10.1016/j.vetmic.2010.03.019 20395076

[B15] Eckert C. Gautier V. Saladin-Allard M. Hidri N. Verdet C. Ould-Hocine Z. (2004). Dissemination of CTX-M-type beta-lactamases among clinical isolates of *Enterobacteriaceae* in Paris. *France. Antimicrob Agents Chemother* 48 1249–1255. 15047527 10.1128/AAC.48.4.1249-1255.2004 PMC375249

[B16] Ewers C. Bethe A. Stamm I. Grobbel M. Kopp P. A. Guerra B. (2014). CTX-M-15-D-ST648 *Escherichia coli* from companion animals and horses: another pandemic clone combining multiresistance and extraintestinal virulence? *J Antimicrob Chemother* 69 1224–1230. 10.1093/jac/dkt516 24398338

[B17] Falgenhauer L. Imirzalioglu C. Ghosh H. Gwozdzinski K. Schmiedel J. Gentil K. (2016). Circulation of clonal populations of fluoroquinolone-resistant CTX-M-15-producing *Escherichia coli* ST410 in humans and animals in Germany. *Int J Antimicrob Agents* 47 457–465. 10.1016/j.ijantimicag.2016.03.019 27208899

[B18] Fibke C. D. Croxen M. A. Geum H. M. Glass M. Wong E. Avery B. P. (2019). Genomic epidemiology of major extraintestinal pathogenic *Escherichia coli* lineages causing urinary tract infections in young women across Canada. *Open Forum Infect Dis* 6 ofz431. 10.1093/ofid/ofz431 31696141 PMC6824535

[B19] Franz E. Veenman C. Van Hoek A. H. De Roda Husman A. Blaak H. (2015). Pathogenic *Escherichia coli* producing extended-spectrum beta-lactamases isolated from surface water and wastewater. *Sci Rep* 5 14372. 10.1038/srep14372 26399418 PMC4585870

[B20] Fujioka M. Kasai K. Miura T. Sato T. Otomo Y. (2009). Rapid diagnostic method for the detection of diarrheagenic *Escherichia coli* by multiplex PCR. *Jpn J Infect Dis* 62 476–480. 19934545

[B21] Gronthal T. Osterblad M. Eklund M. Jalava J. Nykasenoja S. Pekkanen K. (2018). Sharing more than friendship - transmission of NDM-5 ST167 and CTX-M-9 ST69 *Escherichia coli* between dogs and humans in a family, Finland, 2015. *Euro Surveill* 23 1700497. 10.2807/1560-7917.ES.2018.23.27.1700497 29991384 PMC6152158

[B22] Harwalkar A. Gupta S. Rao A. Srinivasa H. (2014). Lower prevalence of *hlyD*, *papC* and *cnf-1* genes in ciprofloxacin-resistant uropathogenic *Escherichia coli* than their susceptible counterparts isolated from southern India. *J Infect Public Health* 7 413–419. 10.1016/j.jiph.2014.04.002 24861644

[B23] Hashemizadeh Z. Kalantar-Neyestanaki D. Mansouri S. (2017). Association between virulence profile, biofilm formation and phylogenetic groups of *Escherichia coli* causing urinary tract infection and the commensal gut microbiota: A comparative analysis. *Microb Pathog* 110 540–545. 10.1016/j.micpath.2017.07.046 28760455

[B24] Hertz F. B. Nielsen J. B. Schonning K. Littauer P. Knudsen J. D. Lobner-Olesen A. (2016). Population structure of drug-susceptible,-resistant and ESBL-producing *Escherichia coli* from community-acquired urinary tract. *BMC Microbiol* 16:63. 10.1186/s12866-016-0681-z 27067536 PMC4827192

[B25] Johnson J. R. (2003). Microbial virulence determinants and the pathogenesis of urinary tract infection. *Infect Dis Clin North Am* 17 261–278. 12848470 10.1016/s0891-5520(03)00027-8

[B26] Johnson J. R. Murray A. C. Gajewski A. Sullivan M. Snippes P. Kuskowski M. A. (2003). Isolation and molecular characterization of nalidixic acid-resistant extraintestinal pathogenic *Escherichia coli* from retail chicken products. *Antimicrob Agents Chemother* 47 2161–2168. 12821463 10.1128/AAC.47.7.2161-2168.2003 PMC161843

[B27] Johnson J. R. Russo T. A. (2005). Molecular epidemiology of extraintestinal pathogenic (uropathogenic) *Escherichia coli*. *International Journal of Medical Microbiology* 295 383–404. 16238015 10.1016/j.ijmm.2005.07.005

[B28] Johnson T. J. Debroy C. Belton S. Williams M. L. Lawrence M. Nolan L. K. (2010). Pyrosequencing of the Vir plasmid of necrotoxigenic *Escherichia coli*. *Vet Microbiol* 144 100–109. 10.1016/j.vetmic.2009.12.022 20060660

[B29] Lau S. H. Reddy S. Cheesbrough J. Bolton F. J. Willshaw G. Cheasty T. (2008). Major uropathogenic *Escherichia coli* strain isolated in the northwest of England identified by multilocus sequence typing. *J Clin Microbiol* 46 1076–1080. 10.1128/JCM.02065-07 18199778 PMC2268376

[B30] LeCuyer T. E. Byrne B. A. Daniels J. B. Diaz-Campos D. V. Hammac G. K. Miller C. B. (2018). Population structure and antimicrobial resistance of canine uropathogenic *Escherichia coli*. *J Clin Microbiol* 56 10.1128/JCM.00788-18 e00788-18 29997200 PMC6113483

[B31] Liu X. Thungrat K. Boothe D. M. (2015). Multilocus sequence typing and virulence profiles in uropathogenic *Escherichia coli* isolated from cats in the United States. *PLoS One* 10:e0143335. 10.1371/journal.pone.0143335 26587840 PMC4654559

[B32] Liu X. Thungrat K. Boothe D. M. (2016). Occurrence of OXA-48 carbapenemase and other β-lactamase genes in ESBL-producing multidrug resistant *Escherichia coli* from dogs and cats in the United States, 2009-2013. *Frontiers in Microbiology* 7:1057. 10.3389/fmicb.2016.01057 27462301 PMC4939299

[B33] Ljungquist O. Ljungquist D. Myrenas M. Ryden C. Finn M. Bengtsson B. (2016). Evidence of household transfer of ESBL-/pAmpC-producing *Enterobacteriaceae* between humans and dogs - a pilot study. *Infect Ecol Epidemiol* 6 31514. 10.3402/iee.v6.31514 27330043 PMC4916256

[B34] Manges A. R. (2016). *Escherichia coli* and urinary tract infections: the role of poultry-meat. *Clin Microbiol Infect* 22 122–129. 10.1016/j.cmi.2015.11.010 26679924

[B35] Manges A. R. Geum H. M. Guo A. Edens T. J. Fibke C. D. Pitout J. D. D. (2019). Global Extraintestinal Pathogenic *Escherichia coli* (ExPEC) lineages. *Clin Microbiol Rev* 32 1–25. 10.1128/CMR.00135-18 PMC6589867 31189557

[B36] Marques C. Belas A. Franco A. Aboim C. Gama L. T. Pomba C. (2018). Increase in antimicrobial resistance and emergence of major international high-risk clonal lineages in dogs and cats with urinary tract infection: 16 year retrospective study. *J Antimicrob Chemother* 73 377–384. 10.1093/jac/dkx401 29136156 PMC5890753

[B37] Melo L. C. Boisson M. N. Saras E. Medaille C. Boulouis H. J. Madec J. Y. (2017). OXA-48-producing ST372 *Escherichia coli* in a French dog. *J Antimicrob Chemother* 72 1256–1258. 28039279 10.1093/jac/dkw531

[B38] Melo L. C. Haenni M. Saras E. Duprilot M. Nicolas-Chanoine M. H. Madec J. Y. (2019). Emergence of the C1-M27 cluster in ST131 *Escherichia coli* from companion animals in France. *J Antimicrob Chemother* 74 3111–3113. 31299071 10.1093/jac/dkz304

[B39] Paton A. W. Beutin L. Paton J. C. (1995). Heterogeneity of the amino-acid sequences of *Escherichia coli* Shiga-like toxin type-I operons. *Gene* 153 71–74. 7883188 10.1016/0378-1119(94)00777-p

[B40] Peigne C. Bidet P. Mahjoub-Messai F. Plainvert C. Barbe V. Medigue C. (2009). The plasmid of *Escherichia coli* strain S88 (O45:K1:H7) that causes neonatal meningitis is closely related to avian pathogenic *E. coli* plasmids and is associated with high-level bacteremia in a neonatal rat meningitis model. *Infect Immun* 77 2272–2284. 10.1128/IAI.01333-08 19307211 PMC2687354

[B41] Pulss S. Stolle I. Stamm I. Leidner U. Heydel C. Semmler T. (2018). Multispecies and clonal dissemination of OXA-48 carbapenemase in *Enterobacteriaceae* from companion animals in Germany, 2009-2016. *Front Microbiol* 9:1265. 10.3389/fmicb.2018.01265 29963026 PMC6010547

[B42] R Core Team. (2019). *R: A language and environment for statistical computing.* Vienna, Austria: R Foundation for Statistical Computing.

[B43] Reeves P. R. Liu B. Zhou Z. Li D. Guo D. Ren Y. (2011). Rates of mutation and host transmission for an *Escherichia coli* clone over 3 years. *PLoS One* 6:e26907. 10.1371/journal.pone.0026907 22046404 PMC3203180

[B44] Roer L. Overballe-Petersen S. Hansen F. Schonning K. Wang M. Roder B. L. (2018). *Escherichia coli* Sequence Type 410 is causing new international high-risk clones. *mSphere* 3 10.1128/mSphere.00337-18 e00337–18 30021879 PMC6052333

[B45] Shibata N. Kurokawa H. Doi Y. Yagi T. Yamane K. Wachino J.-I. (2006). PCR classification of CTX-M-type beta-lactamase genes identified in clinically isolated gram-negative bacilli in Japan. *Antimicrob Agents Chemother* 50 791–795. 16436748 10.1128/AAC.50.2.791-795.2006 PMC1366867

[B46] Spurbeck R. R. Dinh P. C. Jr. Walk S. T. Stapleton A. E. Hooton T. M. Nolan L. K. (2012). *Escherichia coli* isolates that carry *vat*, *fyuA*, *chuA*, and *yfcV* efficiently colonize the urinary tract. *Infect Immun* 80 4115–4122. 10.1128/IAI.00752-12 22966046 PMC3497434

[B47] Subashchandrabose S. Mobley H. L. T. (2015). Virulence and fitness determinants of uropathogenic *Escherichia coli*. *Microbiol Spectr* 3 1–20. 10.1128/microbiolspec.UTI-0015-2012 PMC4566162 26350328

[B48] Timofte D. Maciuca I. E. Williams N. J. Wattret A. Schmidt V. (2016). Veterinary Hospital Dissemination of CTX-M-15 Extended-Spectrum Beta-Lactamase-Producing *Escherichia coli* ST410 in the United Kingdom. *Microb Drug Resist* 22 609–615. 27314838 10.1089/mdr.2016.0036 PMC5073239

[B49] Toth I. Herault F. Beutin L. Oswald E. (2003). Production of cytolethal distending toxins by pathogenic *Escherichia coli* strains isolated from human and animal sources: establishment of the existence of a new *cdt* variant (Type IV). *J Clin Microbiol* 41 4285–4291. 12958258 10.1128/JCM.41.9.4285-4291.2003 PMC193864

[B50] Ukah U. V. Glass M. Avery B. Daignault D. Mulvey M. R. Reid-Smith R. J. (2018). Risk factors for acquisition of multidrug-resistant *Escherichia coli* and development of community-acquired urinary tract infections. *Epidemiol Infect* 146 46–57. 10.1017/S0950268817002680 29229015 PMC9134527

[B51] Van Hout D. Verschuuren T. D. Bruijning-Verhagen P. C. J. Bosch T. Schurch A. C. Willems R. J. L. (2020). Extended-spectrum beta-lactamase (ESBL)-producing and non-ESBL-producing *Escherichia coli* isolates causing bacteremia in the Netherlands (2014 - 2016) differ in clonal distribution, antimicrobial resistance gene and virulence gene content. *PLoS One* 15:e0227604. 10.1371/journal.pone.0227604 31935253 PMC6959556

[B52] Wagner S. Gally D. L. Argyle S. A. (2014). Multidrug-resistant *Escherichia coli* from canine urinary tract infections tend to have commensal phylotypes, lower prevalence of virulence determinants and ampC-replicons. *Vet Microbiol* 169 171–178. 10.1016/j.vetmic.2014.01.003 24485933 PMC3969583

[B53] Weissman S. J. Johnson J. R. Tchesnokova V. Billig M. Dykhuizen D. Riddell K. (2012). High-resolution two-locus clonal typing of extraintestinal pathogenic *Escherichia coli*. *Appl Environ Microbiol* 78 1353–1360. 10.1128/AEM.06663-11 22226951 PMC3294456

[B54] Yamamoto S. (2007). Molecular epidemiology of uropathogenic *Escherichia coli*. *J Infect Chemother* 13 68–73. 17458672 10.1007/s10156-007-0506-y

[B55] Zogg A. L. Simmen S. Zurfluh K. Stephan R. Schmitt S. N. Nuesch-Inderbinen M. (2018a). High prevalence of Extended-Spectrum beta-Lactamase producing *Enterobacteriaceae* among clinical isolates from cats and dogs admitted to a veterinary hospital in Switzerland. *Front Vet Sci* 5:62. 10.3389/fvets.2018.00062 29662886 PMC5890143

[B56] Zogg A. L. Zurfluh K. Schmitt S. Nuesch-Inderbinen M. Stephan R. (2018b). Antimicrobial resistance, multilocus sequence types and virulence profiles of ESBL producing and non-ESBL producing uropathogenic *Escherichia coli* isolated from cats and dogs in Switzerland. *Vet Microbiol* 216 79–84. 10.1016/j.vetmic.2018.02.011 29519530

