# Supplementary figures and images for: Pathogenic Escherichia coli in Dogs Reveals the Predominance of ST372 and the Human-Associated ST73 Extra-Intestinal Lineages
Source: Front Microbiol. 2020 Apr 21;11:580. doi: 10.3389/fmicb.2020.00580 (PMC7186358; doi:10.3389/fmicb.2020.00580)

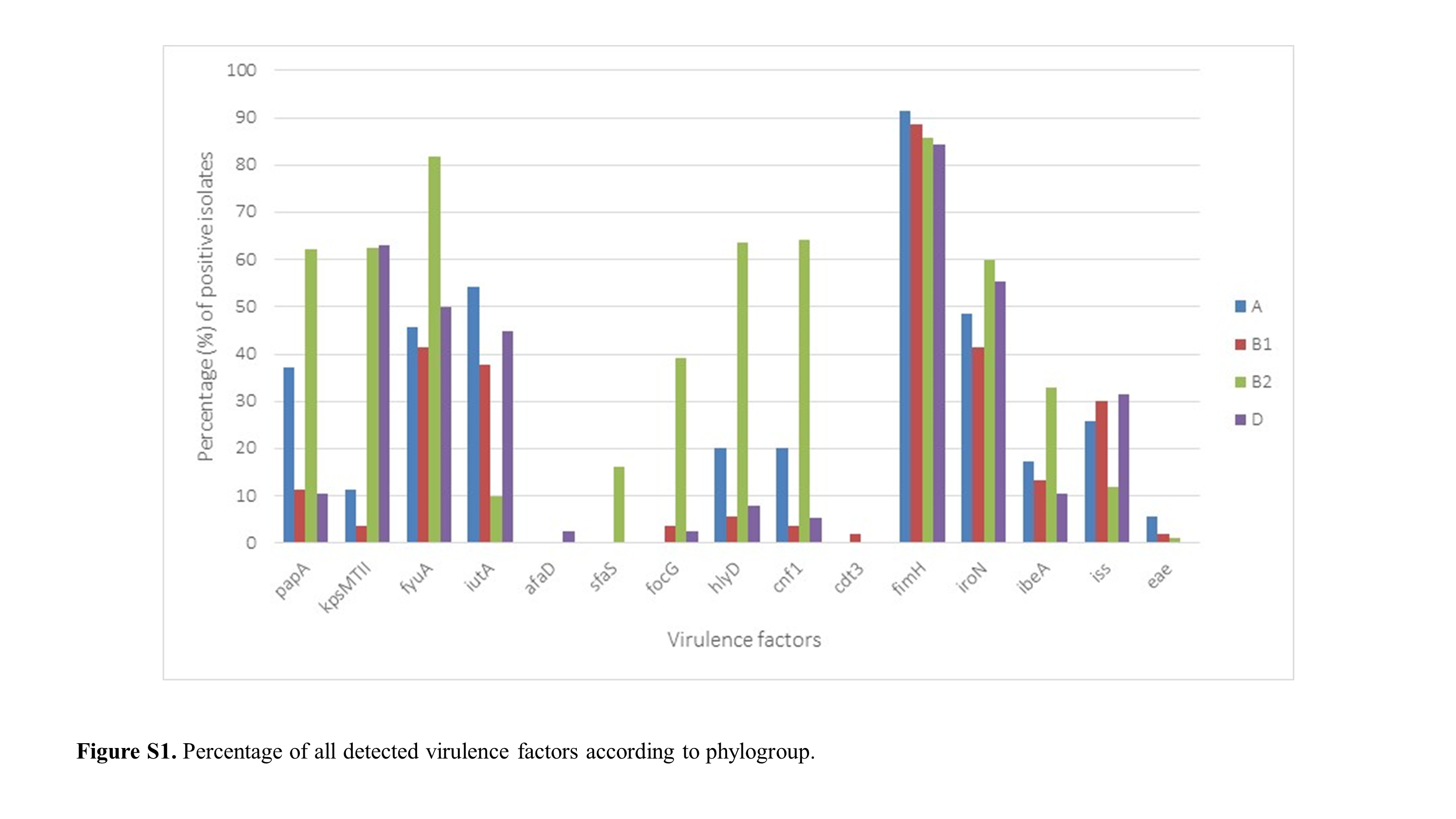

Supplement: Supplementary file 1 [file Image_1.TIF]
