# Supplementary material for: Pathogenic Escherichia coli in Dogs Reveals the Predominance of ST372 and the Human-Associated ST73 Extra-Intestinal Lineages
Source: Front Microbiol. 2020 Apr 21;11:580. doi: 10.3389/fmicb.2020.00580 (PMC7186358; doi:10.3389/fmicb.2020.00580)
Supplement: Supplementary file 2 [file Table_1.docx]

Table S1. Pathologies associated to the 618 *E. coli*.

| Pathologies | Phylogroups | | | | Total |
| --- | --- | --- | --- | --- | --- |
|  | A (%) | B1 (%) | D (%) | B2 (%) |  |
| UTI | 23 (5.7) | 31 (7.7) | 24 (6.0) | 325 (80.6) | 403 (65.2) |
| Respiratory | 0 (0.0) | 2 (11.1) | 1 (5.6) | 15 (83.3) | 18 (2.9) |
| Digestive | 3 (10.3) | 4 (13.8) | 5 (17.2) | 17 (58.6) | 29 (4.7) |
| SSTI^1^ | 2 (7.1) | 1 (3.6) | 2 (7.1) | 23 (82.1) | 28 (4.5) |
| Otitis | 3 (5.0) | 2 (3.3) | 1 (1.7) | 54 (90.0) | 60 (9.7) |
| Reproduction | 2 (6.9) | 3 (10.3) | 0 (0.0) | 24 (8.3) | 29 (4.7) |
| Sepsis | 0 (0.0) | 0 (0.0) | 1 (100) | 0 (0.0) | 1 (0.2) |
| Not available | 2 (4.0) | 10 (20.0) | 4 (8.0) | 34 (68.0) | 50 (80.1) |
| Total | 35 (5.7) | 53 (8.6) | 38 (6.1) | 492 (79.6) | 618 |

^1^SSTI: skin and soft tissue infection
