# Supplementary material for: Pathogenic Escherichia coli in Dogs Reveals the Predominance of ST372 and the Human-Associated ST73 Extra-Intestinal Lineages
Source: Front Microbiol. 2020 Apr 21;11:580. doi: 10.3389/fmicb.2020.00580 (PMC7186358; doi:10.3389/fmicb.2020.00580)
Supplement: Supplementary file 3 [file Table_2.DOCX]

Table S2: Serotypes, acquired virulence and antibiotic resistance genes of 14 uropathogenic *Escherichia coli* (UPEC) isolates

| Strain | Sequence type | Serotype | Virulence genes | Resistance genes |
| --- | --- | --- | --- | --- |
| AA14 | 73 | O6:H1 | *cnf1, gad, iroN, iss, mchB, mchC, mchF, mcmA, pic, vat* | *mdf(A), blaTEM-30, aadA1, sul1, sul2, aph, dfrA1, tet(B)* |
| B08 | 12^1^ | O2:H1 | *cnf1, gad, iha, ireA, iroN, iss, mchB, mchC, mchF, mcmA, pic, sat, sfaS, vat* | *mdf(A)* |
| C20 | 1262^1^ | O15:H31 | *cnf1, gad, iroN, iss, mchB, mchC, mchF, mcmA, vat* | *mdf(A)* |
| D01 | 131 | O4:H5 | *celb, cnf1, gad, ireA, iroN, iss, mchB, mchC, mchF, mcmA, vat* | *mdf(A)* |
| I21 | 372 | O4:H5 | *cnf1, gad, iroN, iss, mchB, mchC, mchF, mcmA, vat* | *mdf(A)* |
| I25 | 141 | O2:H6 | *astA, cnf1, gad, iroN, iss, mchB, mchC, mchF, mcmA, sfaS, vat* | *mdf(A)* |
| I34 | 131 | O25:H4 | *cnf1, gad, iroN, iss, mchB, mchC, mchF, mcmA* | *mdf(A), blaTEM-1B* |
| J19 | 2015 | O2:H14 | *cnf1, gad, iroN, iss, mchB, mchC, mchF, mcmA, vat* | *mdf(A)* |
| J22 | 372 | O4:H31 | *cnf1, gad, iroN, iss, mchB, mchC, mchF, mcmA, vat* | *mdf(A)* |
| N10 | 131 | O4:H5 | *cnf1, gad, ireA, iroN, iss, mchB, mchC, mchF, mcmA, vat* | *mdf(A)* |
| U23 | 141 | O2:H6 | *astA, cnf1, gad, iroN, iss, mchB, mchC, mchF, mcmA, sfaS, vat* | *mdf(A), blaTEM-1B, sul2, aph, dfrA5* |
| V16 | 73 | O6/O4:H1/H5 | *cnf1, gad, ireA, iroN, iss, mchB, mchC, mchF, mcmA, pic, vat* | *mdf(A)* |
| X01 | 73 | O25:H1 | *celb, cnf1, gad, ireA, iroN, iss, mchB, mchC, mchF, mcmA, pic, vat* | *mdf(A)* |
| X21 | 73 | O6:H1 | *cnf1, gad, iha, ireA, iroN, iss, pic, sat, vat* | *mdf(A), blaTEM-1B* |

The serotypes and the identification of acquired virulence and antibiotic resistance genes determined using whole genome analysis are based on Center for Genomic Epidemiology (CGE). <http://www.genomicepidemiology.org/> (accessed on 17 Sept 2019).

^1^. Single locus variant of the given multi-locus sequence type.
