# Supplementary material for: Pathogenic Escherichia coli in Dogs Reveals the Predominance of ST372 and the Human-Associated ST73 Extra-Intestinal Lineages
Source: Front Microbiol. 2020 Apr 21;11:580. doi: 10.3389/fmicb.2020.00580 (PMC7186358; doi:10.3389/fmicb.2020.00580)
Supplement: Supplementary file 4 [file Table_3.DOCX]

Table S3: Frequency of virulence profiles of the 403 uropathogenic *E. coli* (UPEC) isolates

| Profile of virulence | Number of profiles |
| --- | --- |
| *cnf1, focG,, papA,, hlyD,, fimH,, iroN,, ibeA,, fuyA* | 19 |
| *cnf1, kpsMII, papA, hlyD, fimH, iroN, fuyA* | 17 |
| *fimH* | 16 |
| *cnf1, kpsMII, papA, sfaS, hlyD, fimH, iroN, fuyA* | 12 |
| *cnf1, kpsMII, papA, hlyD, fimH, fuyA* | 12 |
| *cnf1, kpsMII, papA, hlyD, fimH, iroN, ibeA, fuyA* | 12 |
| *cnf1, focG, kpsMII, hlyD, fimH, iroN, fuyA* | 10 |
| *cnf1, focG, papA, hlyd, fimH, iroN, fuyA* | 10 |
| *cnf1, focG, kpsMII, papA, hlyD, fimH, iroN, fuyA* | 8 |
| *kpsMII, fimH, fuyA* | 7 |
| *cnf1, papA, hlyD, fimH, iroN, fuyA* | 7 |
| *focG, fimH, iroN, fuyA* | 6 |
| *fimH, fuyA* | 6 |
| *fimH, iroN, iss,* | 5 |
| *iutA, fimH, iroN, iss, fuyA* | 5 |
| *cnf1, focG, kpsMII, papA, hlyD, iutA, fimH, iroN, fuyA* | 5 |
| *kpsMII,* | 4 |
| *kpsMII, fimH, iroN, ibeA, fuyA* | 4 |
| *kpsMII, fimH, ibeA, fuyA* | 4 |
| *cnf1, focG, kpsMII, papA, hlyD, fimHfuyA* | 4 |
| *fimH, ibeA* | 4 |
| *focG, fimH, iroN, ibeA, fuyA* | 4 |
| *cnf1, focG, papA, hlyd, fimH, fuyA* | 4 |
| *cnf1, papA, hlyD, iutA, fimH, fuyA* | 4 |
| *cnf1, papA, hlyD, fimH, fuyA* | 4 |
| *cnf1, kpsMII, papA, sfaS, hlyD, fimH, iroN, ibeA, fuyA* | 4 |
| No virulence gene | 4 |
| *cnf1, papA, hlyD, fimH, iroN, ibeA, fuyA* | 3 |
| *cnf1, kpsMII, papA, sfaS, hlyD, fimH, iroNiss, fuyA* | 3 |
| *cnf1, kpsMII, papA, hlyD, fimH, ibeA, fuyA* | 3 |
| *kpsMII, fimH, iroN, iss, fuyA* | 3 |
| *cnf1, focG, kpsMII, hlyD, fimH, fuyA* | 3 |
| *focG* | 3 |
| *cnf1, kpsMII, papA, hlyD, fimH, iss, fuyA* | 3 |
| *focG, fimH, ibeA, fuyA* | 3 |
| *cnf1, kpsMII, papA, sfaS, hlyD, fimH, fuyA* | 3 |
| *iutA, fimH, fuyA* | 3 |
| *cnf1, focG, papA, sfaS, hlyD, fimH, iroN, ibeA, fuyA* | 3 |
| *focG, kpsMII,* | 3 |
| *cnf1, papA, hlyD, fimH, iroN,* | 3 |
| *papA, iutA, fimH, iroN, iss, fuyA* | 3 |
| *cnf1, kpsMII, papA, hlyD, fimH, iroN, iss, fuyA* | 2 |
| *cnf1, focG, papA, hlyd, fimH, ibeA, fuyA* | 2 |
| *focG, ibeA* | 2 |
| *focG, papA, fimH, fuyA* | 2 |
| *kpsMII, iutA, fimH* | 2 |
| *kpsMII, sfaS, fimH, iroN, fuyA* | 2 |
| *cnf1, kpsMII, papA, hlyD, iutA, fimHfuyA* | 2 |
| *focG, kpsMII, fimH, fuyA* | 2 |
| *cnf1, kpsMII, papA, sfaS, hlyD, iutA, fimH, iroN, ibeA, fuyA* | 2 |
| *focG, kpsMII, fimH, ibeA, fuyA* | 2 |
| *cnf1, focG, papA, sfaS, hlyD, fimH, fuyA* | 2 |
| *focG, kpsMII, fimH, iroN, fuyA* | 2 |
| *kpsMII, papA, iutA,* | 2 |
| *cnf1, focG, kpsMII, papA, hlyD, fimH, iroN, ibeA, fuyA* | 2 |
| *cnf1, kpsMII, papA, hlyD, fimH, iroN, ibeA, iss, fuyA* | 2 |
| *fimH, iroN, ibeA* | 2 |
| *fimH, iroN, ibeA, fuyA* | 2 |
| *kpsMII, sfaS, fimH, iroN, ibeA, fuyA* | 2 |
| *cnf1, kpsMII, papA, sfaS, hlyD, iutA, fimH, iroN, fuyA* | 2 |
| *cnf1, papA, hlyD, fimH, iroN, iss, fuyA* | 2 |
| *cnf1, focG, hlyD, fimH, iroN, ibeA, fuyA* | 2 |
| *kpsMII, papA, ibeA* | 2 |
| *fimH, iroN, fuyA* | 2 |
| *cnf1, papA, hlyD, iutA, fimH, iroN, fuyA* | 2 |
| *focG, fimH, fuyA* | 2 |
| *cnf1, papA, hlyD, iutA, fimH, iroN, iss, fuyA* | 2 |
| *kpsMII, iutA, fimH, fuyA* | 2 |
| *kpsMII, papA, fimH, fuyA* | 2 |
| *papA, iutA,* | 2 |
| *cnf1, papA, hlyD, fimH, iss, fuyA* | 2 |
| *kpsMII, fimH, iroN, fuyA* | 2 |
| *kpsMI, IiutA, fimH, iroN, iss, fuyA* | 1 |
| *papA, iutA, iss,* | 1 |
| *cnf1, focG, kpsMII, papA, hlyD, iroN, fuyA* | 1 |
| *focG, papA, fimH, iroN, ibeA, fuyA* | 1 |
| *focG, fimH* | 1 |
| *focG, papA, hlyD, ibeA* | 1 |
| *focG, fimH, iroN, iss, fuyA* | 1 |
| *focG, papA, sfaS, ibeA* | 1 |
| *cnf1, focG, kpsMII, papA, sfaS, hlyD, fimH, iroN, fuyA* | 1 |
| *kpsMII, sfaS, fimH, fuyA* | 1 |
| *cnf1, kpsMII, papA, sfaS, hlyD, iutA, fimH, fuyA* | 1 |
| *focG, kpsMII, iroN,* | 1 |
| *cnf1, focG, papA, fimH, iroNiss, fuyA* | 1 |
| *kpsMII, iutA, iroN,* | 1 |
| *kpsMII, ibeA* | 1 |
| *kpsMII, fimH, iroN, iss,* | 1 |
| *cnf1, focG, kpsMII, hlyD, fimH, iroN,* | 1 |
| *focG, kpsMII, fimH, iroN,* | 1 |
| *kpsMII, iutA, fimH, iroN, ibeA, fuyA* | 1 |
| *kpsMII, papA* | 1 |
| *cnf1, focG, kpsMII, papA, sfaS, hlyD, iutA, fimH, fuyA* | 1 |
| *focG, kpsMII, fimH, iroN, ibeA, fuyA* | 1 |
| *kpsMII, iutA, fimH, iroN, iss,* | 1 |
| *focG, kpsMII, hlyD, fimH, iss, fuyA* | 1 |
| *focG, iroN,* | 1 |
| *kpsMII, iutA, ibeA, fuyA* | 1 |
| *cnf1, focG, hlyD, fimH, ibeA, fuyA* | 1 |
| *iutA, fimH, iss,* | 1 |
| *sfaS,* | 1 |
| *kpsMII, iutA, fimH, ibeA, fuyA* | 1 |
| *focG, fimH, iroN,* | 1 |
| *cnf1, papA, hlyD, fimH, ibeA, fuyA* | 1 |
| *cnf1, focG, papA, sfaS, hlyD, fimH, ibeA, fuyA* | 1 |
| *kpsMII, papA, fimH, ibeA, fuyA* | 1 |
| *focG, fimH, iroN, ibeA* | 1 |
| *iutA, fimH, iss, fuyA* | 1 |
| *cnf1, focG, kpsMII, hlyD, fimH, iroN, iss, fuyA* | 1 |
| *kpsMII, papA, fimH, iroN, ibeA, fuyA* | 1 |
| *cnf1, focG, kpsMII, papA, hlyD, fimH, iroN,* | 1 |
| *kpsMII, iutA, fimH, iroN,* | 1 |
| *cnf1, focG, kpsMII, papA, sfaS,* | 1 |
| *cnf1, papA, hlyD, iutA, fuyA* | 1 |
| *cnf1, focG, kpsMII, papA, sfaS, hlyD, iutAiroN,* | 1 |
| *iutA, fimH, ibeA, fuyA* | 1 |
| *kpsMII, fimH, iroN,* | 1 |
| *cnf1, papA, hlyD, iutA, fimH, ibeA, fuyA* | 1 |
| *iutA, fimH, iroN, ibeA* | 1 |
| *kpsMII, papA, iutA, fimH* | 1 |
| *iutA, fimH, iroN, ibeA, fuyA* | 1 |
| *kpsMII, papA, iutA, fimH, fuyA* | 1 |
| *cnf1, kpsMII, papA, sfaS, hlyD, iutA, fimH, iss, fuyA* | 1 |
| *papA, iutA, fimH, iss, fuyA* | 1 |
| *cnf1, kpsMII, papA, sfaS, hlyD, iutA, fimH, iroNiss, fuyA* | 1 |
| *cnf1, kpsMII, sfaS, hlyD, iutA, fimH, iroNiss, fuyA* | 1 |
| *cnf1, kpsMII, papA, sfaS, hlyD, iutA, fimH, iroN, ibeA, iss,* | 1 |
| *cnf1, kpsMII, papA, fimH, iroN, ibeAfuyA* | 1 |
| *kpsMII, iutA, fimH, iroN, iss, fuyA* | 1 |
| *cnf1, kpsMII, papA, hlyD, ibeA, iss, fuyA* | 1 |
| *cnf1, focG, papA, hlyD, ibeA* | 1 |
| *cnf1, kpsMII, papA, hlyD, iroN, fuyA* | 1 |
| *cnf1, focG, papA, hlyd, fimH, iss, fuyA* | 1 |
| *cnf1, kpsMII, papA, hlyD, iroN, ibeA, fuyA* | 1 |
| *cnf1, focG, papA, hlyd, fimH, iroN,* | 1 |
| *cnf1, kpsMII, papA, hlyD, fimH* | 1 |
| *cnf1, focG, papA, hlyd, fimH, iroN, iss, fuyA* | 1 |
| *iutA, fimH, iroN,* | 1 |
| *fimH, iroN, ibeA, iss,* | 1 |
| *cnf1, kpsMII, papA, hlyD, fimH, iss,* | 1 |
| *cnf1, focG, papA, sfaS, hlyD, fimH, iroN, fuyA* | 1 |
| *kpsMII, papA, hlyD, ibeA* | 1 |
| *cnf1, focG, kpsMII, hlyD, iroN, fuyA* | 1 |
| *cnf1, kpsMII, papA, hlyDfimH, iss, fuyA* | 1 |
| *cnf1, focG, kpsMII, hlyD, fimH, ibeA, fuyA* | 1 |
| *kpsMII, papA, hlyD, fimH, fuyA* | 1 |
| *kpsMII, iroN,* | 1 |
| *cnf1, kpsMII, papA, hlyD, fimH, ibeA, iss, fuyA* | 1 |
| *cnf1, focG, kpsMII, hlyD, fimH, iroN, ibeA, iss, fuyA* | 1 |
| *iutA, fimH, iroN, iss,* | 1 |
| *ibeA* | 1 |
| *kpsMII, papA, hlyD, iutA, iroN, ibeA, fuyA* | 1 |
| *kpsMII, fimH, iss, fuyA* | 1 |
| *fimH, iroN,* | 1 |
| *focG, papA* | 1 |
| *kpsMII, papA, sfaS, iroN,* | 1 |
| *cnf1, focG, kpsMII, papA, sfaS, hlyD, fimH, fuyA* | 1 |
| *kpsMII, papA, sfaS, iutA,* | 1 |
| *cnf1, focG, kpsMII, papA, sfaS, hlyD, fimH, iroN, ibeA, fuyA* | 1 |
| *cnf1, kpsMII, papA, hlyD, iutA, fimH, iroN, fuyA* | 1 |
| *cnf1, focG, kpsMII, papA, sfaS, hlyD, iutA, fimH* | 1 |
| *cnf1, kpsMII, papA, sfaS, fimH, iroN, ibeA, fuyA* | 1 |
| *cnf1, focG, kpsMII, papA, sfaS, hlyD, iutA, fimH, iroN, fuyA* | 1 |
| *cnf1, kpsMII, papA, sfaS, hlyD, fimH, fuyA* | 1 |
| *focG, papA, ibeA* | 1 |
| *kpsMIIiutA, fimH, iroN, fuyA* | 1 |
| *kpsMII, sfaS,* | 1 |
| *cnf1, kpsMII, papA, sfaS, hlyD, fimH, iroN,* | 1 |
| *cnf1, kpsMII, hlyD, fimH, iroN, ibeA, fuyA* | 1 |
| *focG, kpsMII, papA, sfaS, iutA, fimH, iroN,* | 1 |
| Total | **403** |

,
